# Wheat powdery mildew resistance: from gene identification to immunity deployment

**DOI:** 10.3389/fpls.2023.1269498

**Published:** 2023-09-18

**Authors:** Shenghao Zou, Yang Xu, Qianqian Li, Yali Wei, Youlian Zhang, Dingzhong Tang

**Affiliations:** State Key Laboratory of Ecological Control of Fujian-Taiwan Crop Pests, Key Laboratory of Ministry of Education for Genetics, Breeding and Multiple Utilization of Crops, Plant Immunity Center, Fujian Agriculture and Forestry University, Fuzhou, China

**Keywords:** powdery mildew, *Blumeria graminis* f. sp. *tritici*, wheat, NLR, race-specific resistance

## Abstract

Powdery mildew is one of the most devastating diseases on wheat and is caused by the obligate biotrophic phytopathogen *Blumeria graminis* f. sp. *tritici* (*Bgt*). Due to the complexity of the large genome of wheat and its close relatives, the identification of powdery mildew resistance genes had been hampered for a long time until recent progress in large-scale sequencing, genomics, and rapid gene isolation techniques. Here, we describe and summarize the current advances in wheat powdery mildew resistance, emphasizing the most recent discoveries about the identification of genes conferring powdery mildew resistance and the similarity, diversity and molecular function of those genes. Multilayered resistance to powdery mildew in wheat could be used for counteracting *Bgt*, including durable, broad spectrum but partial resistance, as well as race-specific and mostly complete resistance mediated by nucleotide-binding and leucine rich repeat domain (NLR) proteins. In addition to the above mentioned layers, manipulation of susceptibility (S) and negative regulator genes may represent another layer that can be used for durable and broad-spectrum resistance in wheat. We propose that it is promising to develop effective and durable strategies to combat powdery mildew in wheat by simultaneous deployment of multilayered immunity.

## Introduction

Bread wheat (*Triticum aestivum* L.) is the most widely cultivated crop worldwide, providing approximately 20% of the total daily calories consumed by humans (Reynolds et al., 2012; Appels et al., 2018). Wheat powdery mildew, caused by *Blumeria graminis* f. sp. *tritici* (*Bgt*), is highly destructive, resulting in nearly 5% of the annual wheat yield loss globally (Singh et al., 2016; Savary et al., 2019). To combat this disease, researchers have cataloged over 100 powdery mildew (Pm) resistance alleles at nearly 65 loci in bread wheat and its relatives (**Table S1**) (Mapuranga et al., 2022). Due to recent progress in large-scale genomic sequencing alongside innovative gene cloning strategies, more powdery mildew resistance genes have been cloned, broadening the disease resistance diversity in wheat.

**Figure 1:**
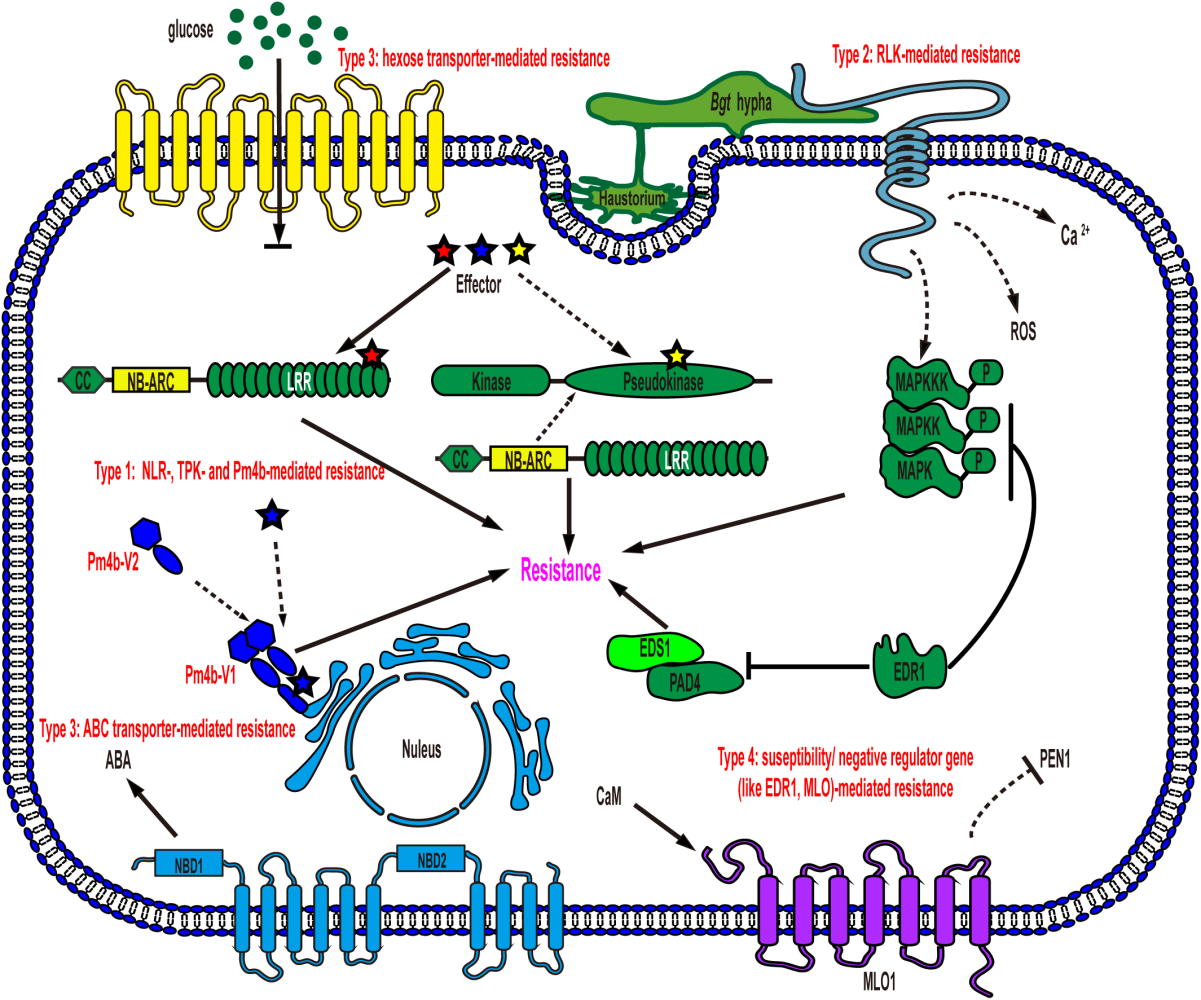
Schematic representation of models for wheat powdery mildew resistance gene function in an epidermal cell. Type 1: NLR-, TPK- and Pm4b-mediated resistance. NLRs and several non-NLRs, such as TPK and Pm4b, mediate race-specific resistance after recognizing cognate effectors secreted by *Bgt*, leading to the induction of cell death and resistance to powdery mildew. TKP: tandem kinase proteins; Type 2: RLK-mediated resistance. Plasma membrane-localized RLKs detect PAMPs of *Bgt*, triggering downstream signaling events such as mitogen-activated protein kinase (MAPK) cascades, calcium (Ca^2+^) flux, and reactive oxygen species (ROS) bursts, ultimately providing defense against powdery mildew; Type 3: hexose transporter- and ABC transporter-mediated resistance. Membrane-localized transporters create a hostile environment for pathogen growth, resulting in resistance without requiring specific recognition of *Bgt*. NBD: nucleotide binding domain; Type 4: susceptibility/negative regulator gene-mediated resistance. Loss-of-function mutations in these genes, such as *EDR1* and *MLO*, enhance powdery mildew resistance in wheat. EDR1 negatively regulates key components of plant innate immunity, including MAPK, EDS1 and PAD4. MLO, on the other hand, modulates PEN1-associated vesicle fusion and exocytosis processes to support pathogenesis.

To date, most of the identified wheat powdery mildew resistance genes encode CNL proteins with nucleotide-binding sites (NBS) and leucine-rich repeat (LRR) domains (NLR) associated with coiled-coils at the N-termini, such as Pm1a, Pm2, Pm3/Pm8/Pm17, Pm5e, Pm21/Pm12, Pm41, Pm60 and Pm69, which perceive the effectors secreted by *Bgt*, conferring effector triggered immunity (ETI) and showing race-specific resistance (Gupta et al., 2022). Race-specific resistance can mostly provide complete resistance to specific *Bgt* isolates when CNL immune receptors and their cognate effectors, encoded by pathogen avirulence (*Avr*) genes, are present. However, the gene-specific arms race, causing diversification of both immune receptor and Avr genes, can overcome this type of resistance rapidly, especially in wheat with a single race-specific immune receptor gene (Brunner et al., 2011). Apart from the mentioned CNL receptors, recently, two types of non-NLR immune receptors have also been reported to trigger race-specific resistance. (Lu et al., 2020; Sánchez-Martín et al., 2021; Gaurav et al., 2022). These non-NLR immune receptors contain (pseudo)kinase domains that may be responsible for detecting invading effectors (Sánchez-Martín and Keller, 2021). However, the cognate Avr effectors of these non-NLR immune receptors have not yet been identified. This discovery suggests that not only the typical intracellularly localized NLR proteins but also some non-NLR receptors may play a significant role in activating race-specific resistance against *Bgt* (**Figure 1**, Type 1).

Pattern-triggered immunity (PTI), in contrast to ETI, is the other tiered innate immune system that is activated by the recognition of pathogen-/damage-derived molecules via cell surface-localized pattern-recognition receptors (PRRs) (Jones and Dangl, 2006). PRRs mainly include receptor-like kinases (RLKs) and receptor-like proteins (RLPs). RLKs, found in plants, are a diverse family of proteins with an ectodomain (ECD), a single-pass transmembrane domain, and a cytoplasmic kinase domain, while RLPs lack the intracellular kinase domain (Tang et al., 2017). The ECDs of RLKs and RLPs are highly variable, encompassing leucine-rich repeat (LRR) domains, lysine motifs (LysMs), lectin domains, malectin-like domains, epidermal growth factor (EGF)-like domains, and others, allowing them to recognize a wide range of ligands, including steroids, peptides, polysaccharides, and lipopolysaccharides (Tang et al., 2017). Recently, several types of RLKs (such as TaRLK, LecRK-V, TtdLRK10L-1, HvLEMK1, and RLK-V) that trigger resistance in wheat have been identified, but the corresponding pathogen-associated molecular patterns (PAMPs) or ligands are yet to be identified. However, their conferred powdery mildew resistance is believed to be mainly activated through the recognition of the *Bgt*-associated molecular pattern (**Figure 1**, Type 2).

Unlike resistance genes that activate ETI or PTI, certain wheat powdery mildew resistance genes, such as *Pm38* and *Pm46*, do not require the perception of *Bgt* (**Figure 1**, Type 3). Intriguingly, both *Pm38* and *Pm46* encode membrane-localized transporter proteins (Krattinger et al., 2009; Moore et al., 2015). The powdery mildew resistance, conferred by either PTI or these two transporters, is mostly partial and quantitative, in contrast to the race-specific complete resistance. Notably, partial and quantitative resistance tends to be durable and broad-spectrum, providing protection against all races of the pathogen species (Moore et al., 2015; Sánchez-Martín and Keller, 2021).

In addition to the three types of resistance genes mentioned above, susceptibility genes such as *Mlo* and negative regulator genes such as *EDR1* provide a distinct form of powdery mildew resistance through loss-of-function, which is recessively inherited, with varying levels of resistance depending on the specific susceptibility gene’s function (**Figure 1**, Type 4) (Wang et al., 2014; Zhang et al., 2017; Sánchez-Martín and Keller, 2021). MLO is a membrane-localized protein, while EDR1 is a cytoplasmic Raf-like mitogen-activated protein kinase (MAPK) kinase kinase (MAPKKK) (Büschges et al., 1997; Frye et al., 2001). Each of them plays a distinct role in wheat cell signaling pathways, and the extent of resistance conferred by knocking out them is determined by their intrinsic physiological and biochemical functions in plants. Overall, multilayered resistance to wheat powdery mildew has been identified in recent studies, and combining all the layers simultaneously and strategically would shed light on wheat resistance breeding, even though the molecular mechanism underlying some of the resistance remains to be fully understood.

## The phytopathogen causing wheat powdery mildew

*Blumeria graminis*, a fungal plant pathogen, is responsible for powdery mildew (Savary et al., 2019). Based on their host specificity, multiple formae speciales are defined within the species, such as *Blumeria graminis* f. sp. *tritici* (*Bgt*), which specifically infects wheat and causes wheat powdery mildew. For typical images of the disease and pathogen, readers are referred to a previous review (Jankovics et al., 2015). To survive harsh conditions, *Bgt* can form chasmothecia and undergo a sexual life cycle (Jankovics et al., 2015). *Bgt* originated in the Fertile Crescent during wheat domestication, and historical human migration and trade facilitated its worldwide spread (Sotiropoulos et al., 2022). For the global races of *Bgt* and their spread, readers are referred to a recent review (Sotiropoulos et al., 2022). The sequencing and assembly of the 166 Mb *Bgt* genome, consisting of 11 chromosomes, revealed approximately 844 candidate effector genes (Müller et al., 2018). Hybridization of globally spread mildew isolates has further complicated the genome, accelerating adaptation to new wheat hosts (Müller et al., 2021).

## NLR and several Non-NLR immune receptors activate race-specific resistance via effector recognition

An increasing number of race-specific resistance genes, providing efficient resistance to the rapidly evolving *Bgt*, have been identified in wheat and its wild relatives (**Table 1**). During molecular identification, most of the cloned race-specific resistance genes encode CNL immune receptors, with some identified as orthologous genes, such as *Pm12/Pm21* and *Pm8/Pm3* (Hurni et al., 2013; Zhu et al., 2023). *Pm60*, *Pm60a* and *Pm60b* are functional allelic variants found in different *Triticum urartu* accessions. Compared with *Pm60*, *Pm60b* contains a 240-nucleotide insertion, and its protein has two additional LRR motifs. In contrast, *Pm60a* has a 240-nucleotide deletion and two fewer LRR motifs in its protein, which narrows its *Bgt* resistance spectrum, whereas insertion of the two LRR motifs in Pm60b has comparatively little influence (Zou et al., 2022). Pm3b, Pm3a and Pm3d proteins are also allelic variants differing by a few amino acid (aa) point mutations, mainly in the NBS and LRR domains, which are responsible for recognizing different *Bgt* strains. The cognate Avr effectors of Pm3a, Pm3b and Pm3d were isolated, which belong to a large group of proteins with low sequence homology but predicted structural similarity (Bourras et al., 2019). In addition to *AvrPm3b*, *c*, and *d*, several other avirulence genes have been isolated (**Table 1**). However, the identification of *Bgt* avirulence effectors and the downstream pathways following perception still lags behind resistance gene cloning. Nonetheless, it is believed that there might be a direct interaction between CNLs and their corresponding effectors, resulting in the activation of the hypersensitive response and resistance, as seen with Pm1a and Pm3b (Bourras et al., 2015; Bourras et al., 2019).

**Table 1:** List of cloned genes conferring race-specific resistance to *Blumeria graminis* f. sp. *tritici* in wheat and their corresponding avirulence (*Avr*) genes cloned in the pathogens.

Gene	Gene Product	Donor species	Cognate *Avr*
*Pm1a* (Hewitt et al., 2021)	CNL ^a^	*Triticum aestivum*	*AvrPm1a.1, AvrPm1a.2* (Hewitt et al., 2021; Kloppe et al., 2023)
*Pm2* (Sanchez-Martin et al., 2016)	CNL	*Aegilops tauschii*	*AvrPm2* (Praz et al., 2017; Manser et al., 2021)
*Pm3b* (Yahiaoui et al., 2004)	CNL	*Triticum aestivum*	*AvrPm3b* (Bourras et al., 2019)
*Pm3a, d* (Srichumpa et al., 2005)	CNL	*Triticum aestivum*	*AvrPm3a, d* (Bourras et al., 2015; Bourras et al., 2019)
*Pm5e* (Xie et al., 2020)	CNL	*Triticum aestivum*	–
*Pm8* (Hurni et al., 2013)	CNL	*Secale cereale*	–
*Pm12* (Zhu et al., 2023)	CNL	*Aegilops speltoides*	–
*Pm17* (Singh et al., 2018)	CNL	*Secale cereale*	*AvrPm17* (Müller et al., 2022)
*Pm21* (He et al., 2018; Xing et al., 2018)	CNL	*Dasypyrum villosum*	–
*Pm41* (Li et al., 2020)	CNL	*Triticum turgidum ssp. dicoccoides*	–
*Pm60, 60a, 60b* (Zou et al., 2018)	CNL	*Triticum urartu*	–
*Pm69* (Kim et al., 2023)	CNL	*Triticum turgidum ssp. dicoccoides*	–
*Pm24* (Lu et al., 2020)	TPK ^b^	*Triticum aestivum*	–
*WTK4* ^c^ (Gaurav et al., 2022)	TPK	*Aegilops tauschii*	–
*Pm4b* (Sánchez-Martín et al., 2021)	MCTP kinase ^d^	*Triticum carthlicum*	–

a CNL, coiled-coil (CC), nucleotide binding site (NBS), leucine rich repeat (LRR) protein.

b TKP, tandem kinase protein.

c WTK, wheat tandem kinase.

d MCTP kinase, multiple C2-domains and transmembrane region kinase protein. "-" is that no cognate gene has been reported there.

Pm24 and WTK4 are tandem kinase proteins (TKP) composed of two tandem kinase domains, one of them is a pseudokinase domain, which are reported to confer resistance to *Bgt* (**Table 1**) (Lu et al., 2020; Gaurav et al., 2022). In addition to *Pm24* and *WTK4*, the barley stem rust resistance gene *Rpg1* and the wheat yellow rust resistance gene *Yr15* encode TKPs as well, which have been considered cytosolic localized and race-specific (Brueggeman et al., 2002; Klymiuk et al., 2018). Thus, although Pm24 is resistant to all of the tested 93 *Bgt* isolates collected from China and no virulent isolates have been discovered, by now, it suggests that Pm24 and WTK4 might confer race-specific resistance, similar to other reported TKPs. Furthermore, it has been hypothesized that the pseudokinase domain in these TKPs is the target of *Bgt* effectors and that the interaction activates either TKPs to phosphorylate downstream components or NLRs that guard the TKPs, consequently resulting in resistance (Sánchez-Martín and Keller, 2021; Fahima and Coaker, 2023).

*Pm4b* is a novel race-specific wheat powdery mildew resistance gene (**Figure 1**, Type 1). Functional analysis has revealed that both protein variants resulting from alternative splicing are essential for the resistance function. These two protein isoforms share a kinase domain with serine/threonine specificity. However, in their C-terminus, one isoform has a single C2C domain, while the other contains a C2D domain coupled to a phosphoribosyl transferase C-terminal domain with two transmembrane domains. (Sánchez-Martín et al., 2021). Moreover, the two protein variants have the ability to form an ER-anchored heterocomplex. In this complex, the C2C/D or kinase domains may recognize the cognate effector, leading to the activation of kinase activity and subsequent disease resistance (Sánchez-Martín et al., 2021). Additionally, several functional allelic variants have been discovered in the *Pm4* locus, suggesting a diverse range of resistance capabilities.

Given the complexity of the enormous genome of wheat and its close relatives, it is predicted that more race-specific resistance genes, whether encoding NLR or non-NLR proteins, will likely be isolated in the future.

## Putative powdery mildew resistance proteins responsible for recognizing PAMPs

Several RLKs were identified as conferring wheat powdery mildew resistance, such as RLK-V and LEMK1 with an LRR-malectin domain, LecRK-V with an L-type lectin domain, and TtdLRK10L-1, TaRLK1, and TaRLK2 with an LRR domain (Chen et al., 2016; Rajaraman et al., 2016; Hu et al., 2018; Wang et al., 2018; Xia et al., 2021). These RLKs may be responsible for recognizing PAMPs, as known in PTI, although the corresponding ligands have not yet been isolated (**Figure 1**, Type 2). Additionally, RLK-V has been shown to be required for resistance mediated by Pm21, supporting the model that ETI and PTI mutually potentiate and interdepend on each other (Yuan et al., 2021).

## The powdery mildew resistance proteins that function independently of perceiving *Bgt*

Pm38 and Pm46 are two powdery mildew resistance proteins that confer protection against the pathogen without directly perceiving effectors or PAMPs originating from *Bgt* (**Figure 1**, type 3). Pm38 shares structural similarities with adenosine triphosphate-binding cassette (ABC) transporters of the pleiotropic drug resistance subfamily, which includes the well-known nonhost resistance protein PEN3 in Arabidopsis. Recently, it was reported that abscisic acid (ABA) is the substrate of the ABC transporter Pm38 and that ABA redistribution, mediated via the transporter, might contribute to resistance against not only *Bgt* but also multiple fungal pathogens in wheat (Krattinger et al., 2019; Bräunlich et al., 2021). On the other hand, Pm46 functions as a plasma membrane-localized nonfunctional hexose transporter, leading to an increased hexose/sucrose ratio in the leaf apoplasm due to its blocking of apoplastic hexose retrieval in epidermal cells (**Figure 1**, Type 3)(Moore et al., 2015). Consequently, the altered sugar ratio triggers a sugar-mediated signaling response, creating a more hostile environment for pathogen growth (Proels and Hückelhoven, 2014). Notably, this type of resistance exhibits partial, durable, and broad-spectrum characteristics, similar to PTI, which stands in contrast to race-specific resistance. Remarkably, the perception of *Bgt* is not required for the activation of this type of resistance.

## Modification of susceptibility/negative regulator genes for resistance to wheat powdery mildew

EDR1 is well conserved and is expected to function similarly in different plant species (Frye et al., 2001). In Arabidopsis, EDR1 negatively regulates PTI modulated by the RLP53-associated immune complex (Chen et al., 2022). Notably, EDR1 physically interacts with MKK4/MKK5 and negatively affects MPK3 and MPK6 protein levels and kinase activity (Zhao et al., 2014; Gao et al., 2021). Moreover, EDR1 directly associates with PAD4 and EDS1 and interferes with the heteromeric association of PAD4 and EDS1, which are key components of ETI (Neubauer et al., 2020) (**Figure 1**, Type 4). As a result, EDR1 acts as a negative regulator in innate immunity. Simultaneous modification of three homoeologs of *EDR1* in common wheat via genome editing relieves the suppression of EDR1 to immunity and significantly enhances powdery mildew resistance, although the resistance remains partial (Zhang et al., 2017). *MLO* encodes a transmembrane protein. The C-terminal domain in MLO is responsible for Ca^2+^-dependent binding with calmodulin, which is associated with the negatively regulating ability of MLO to resistance (Kim et al., 2002). Furthermore, resistance mediated by knockout of MLO requires PEN1, suggesting that powdery mildew fungi may enlist MLO to regulate vesicle fusion and exocytosis processes for successful pathogenesis (**Figure 1**, Type 4)(Kusch and Panstruga, 2017; Jacott et al., 2021). Intriguingly, in *mlo* mutants, the development of powdery mildew is terminated at the stage of cell wall penetration, a phenomenon reminiscent of nonhost resistance mechanisms (Jacott et al., 2021). The *mlo* mutants also exhibit undesired pleiotropic phenotypes, including growth penalties and yield losses, which have hampered their widespread use (Consonni et al., 2006). However, a recently identified mutant, with a 304-kilobase pair targeted deletion in the *MLO-B1* locus of wheat, retains crop growth and yields while conferring robust powdery mildew resistance, although the precise mechanism by which to revert the growth penalties is not fully understood (Li et al., 2022; Najafi and Palmgren, 2022). Thus, disruption of this type of gene emerges as an attractive resistance breeding strategy in wheat.

## Prospect

In race-specific powdery mildew resistance, some interactions between NLRs and cognate effectors have been subject to investigation, but the downstream signaling pathways resulting from these interactions remain to be fully understood. In contrast to NLR, the molecular analysis of non-NLR-based race-specific resistance is in its early stages, and the corresponding avirulence genes have not yet been identified. In addition, the ligands recognized by the RLKs for activating PTI to *Bgt* are also unknown. On the other hand, the characterization of resistance conferred by membrane-localized transporters, which do not require the perception of effectors or PAMPs from *Bgt*, has been more extensively studied. Although the resistance provided by these transporters is partial, similar to PTI triggered by RLKs, it tends to be more durable and broad-spectrum compared to race-specific powdery mildew resistance. Notably, resistance facilitated by genome modification of the susceptibility gene *MLO* is complete, durable, and broad-spectrum. Moreover, the undesirable growth penalties observed in *mlo* mutants can potentially be overcome by implementing additional precision genome editing to stack genetic changes.

Resistance to powdery mildew in wheat can be classified into different layers based on their specific characteristics. However, it is important to note that these layers of resistance should not be used individually to combat rapidly evolving *Bgt*. Studies have shown that even on *mlo* plants, powdery mildew isolates with enhanced virulence can emerge (Schwarzbach, 1979). The combination of multilayered resistance is necessary to create a high barrier, preventing *Blumeria graminis* f. sp. *tritici* from adapting to and overcoming (Dracatos et al., 2023). Thus, constant efforts are required to identify and understand various resistance genes in wheat and its close relatives, as well as analyze their molecular mechanisms. This ongoing research is essential for laying the foundation to develop effective and durable strategies to combat wheat powdery mildew.

## Author contributions

SZ: Writing – original draft. YX: Writing – review & editing. QL: Writing – review & editing. YW: Writing – review & editing. YZ: Writing – review & editing. DT: Writing – review & editing.

## References

[B1] Appels R. Eversole K. Stein N. Feuillet C. Keller B. Rogers J.. (2018). Shifting the limits in wheat research and breeding using a fully annotated reference genome. Science 361 (6403), 1–13. doi: 10.1126/science.aar7191 30115783

[B3] Bourras S. Kunz L. Xue M. Praz C. R. Müller M. C. Kälin C.. (2019). The AvrPm3-Pm3 effector-NLR interactions control both race-specific resistance and host-specificity of cereal mildews on wheat. Nat. Commun. 10 (1), 2292–2292. doi: 10.1038/s41467-019-10274-1 31123263 PMC6533294

[B4] Bourras S. McNally K. E. Ben-David R. Parlange F. Roffler S. Praz C. R.. (2015). Multiple avirulence loci and allele-specific effector recognition control the pm3 race-specific resistance of wheat to powdery mildew. Plant Cell 27 (10), 2991–3012. doi: 10.1105/tpc.15.00171 26452600 PMC4682313

[B5] Bräunlich S. Koller T. Glauser G. Krattinger S. G. Keller B. (2021). Expression of the wheat disease resistance gene Lr34 in transgenic barley leads to accumulation of abscisic acid at the leaf tip. Plant Physiol. Biochem. 166, 950–957. doi: 10.1016/j.plaphy.2021.07.001 34247109

[B6] Brueggeman R. Rostoks N. Kudrna D. Kilian A. Han F. Chen J.. (2002). The barley stem rust-resistance gene Rpg1 is a novel disease-resistance gene with homology to receptor kinases. Proc. Natl. Acad. Sci. U.S.A. 99 (14), 9328–9333. doi: 10.1073/pnas.142284999 12077318 PMC123140

[B7] Brunner S. Hurni S. Herren G. Kalinina O. von Burg S. Zeller S. L.. (2011). Transgenic Pm3b wheat lines show resistance to powdery mildew in the field. Plant Biotechnol. J. 9 (8), 897–910. doi: 10.1111/j.1467-7652.2011.00603.x 21438988

[B2] Büschges R. Hollricher K. Panstruga R. Simons G. Wolter M. Frijters A.. (1997). The barley Mlo gene: a novel control element of plant pathogen resistance. Cell 88 (5), 695–705. doi: 10.1016/s0092-8674(00)81912-1 9054509

[B8] Chen R. Sun P. Zhong G. Wang W. Tang D. (2022). The RECEPTOR-LIKE PROTEIN53 immune complex associates with LLG1 to positively regulate plant immunity. J. Integr. Plant Biol. 64 (9), 1833–1846. doi: 10.1111/jipb.13327 35796320

[B9] Chen T. Xiao J. Xu J. Wan W. Qin B. Cao A.. (2016). Two members of TaRLK family confer powdery mildew resistance in common wheat. BMC Plant Biol. 16, 27. doi: 10.1186/s12870-016-0713-8 26810982 PMC4727334

[B10] Consonni C. Humphry M. E. Hartmann H. A. Livaja M. Durner J. Westphal L.. (2006). Conserved requirement for a plant host cell protein in powdery mildew pathogenesis. Nat. Genet. 38 (6), 716–720. doi: 10.1038/ng1806 16732289

[B11] Dracatos P. M. Lu J. Sánchez-Martín J. Wulff B. B. H. (2023). Resistance that stacks up: engineering rust and mildew disease control in the cereal crops wheat and barley. Plant Biotechnol. J. doi: 10.1111/pbi.14106 PMC10502761 37494504

[B12] Fahima T. Coaker G. (2023). Pathogen perception and deception in plant immunity by kinase fusion proteins. Nat. Genet. 55 (6), 908–909. doi: 10.1038/s41588-023-01396-w 37217715 PMC10754050

[B13] Frye C. A. Tang D. Innes R. W. (2001). Negative regulation of defense responses in plants by a conserved MAPKK kinase. Proc. Natl. Acad. Sci. U.S.A. 98 (1), 373–378. doi: 10.1073/pnas.98.1.373 11114160 PMC14597

[B14] Gao C. Sun P. Wang W. Tang D. (2021). Arabidopsis E3 ligase KEG associates with and ubiquitinates MKK4 and MKK5 to regulate plant immunity. J. Integr. Plant Biol. 63 (2), 327–339. doi: 10.1111/jipb.13007 32877006

[B15] Gaurav K. Arora S. Silva P. Sánchez-Martín J. Horsnell R. Gao L.. (2022). Population genomic analysis of Aegilops tauschii identifies targets for bread wheat improvement. Nat. Biotechnol. 40 (3), 422–431. doi: 10.1038/s41587-021-01058-4 34725503 PMC8926922

[B16] Gupta P. Balyan H. Sharma P. Gaurav s.S. Sharma S. Kumar R.. (2022). Catalogue of gene symbols for wheat: 2022 supplement. Annu. Wheat Newslett. 68, 68–81.

[B17] He H. Zhu S. Zhao R. Jiang Z. Ji Y. Ji J.. (2018). Pm21, encoding a typical CC-NBS-LRR protein, confers broad-spectrum resistance to wheat powdery mildew disease. Mol. Plant 11 (6), 879–882. doi: 10.1016/j.molp.2018.03.004 29567454

[B18] Hewitt T. Müller M. C. Molnár I. Mascher M. Holušová K. Šimková H.. (2021). A highly differentiated region of wheat chromosome 7AL encodes a Pm1a immune receptor that recognizes its corresponding AvrPm1a effector from Blumeria graminis. New Phytol. 229 (5), 2812–2826. doi: 10.1111/nph.17075 33176001 PMC8022591

[B19] Hu P. Liu J. Xu J. Zhou C. Cao S. Zhou W.. (2018). A malectin-like/leucine-rich repeat receptor protein kinase gene, RLK-V, regulates powdery mildew resistance in wheat. Mol. Plant Pathol. 19 (12), 2561–2574. doi: 10.1111/mpp.12729 30030900 PMC6637979

[B20] Hurni S. Brunner S. Buchmann G. Herren G. Jordan T. Krukowski P.. (2013). Rye *Pm8* and wheat *Pm3* are orthologous genes and show evolutionary conservation of resistance function against powdery mildew. Plant J. 76 (6), 957-969. doi: 10.1111/tpj.12345 24124925

[B21] Jacott C. N. Ridout C. J. Murray J. D. (2021). Unmasking mildew resistance locus O. Trends Plant Sci. 26 (10), 1006–1013. doi: 10.1016/j.tplants.2021.05.009 34175219

[B22] Jankovics T. Komáromi J. Fábián A. Jäger K. Vida G. Kiss L. (2015). New Insights into the Life Cycle of the Wheat Powdery Mildew: Direct Observation of Ascosporic Infection in *Blumeria graminis* f. sp. *tritici* . Phytopathology 105 (6), 797–804. doi: 10.1094/PHYTO-10-14-0268-R 25710203

[B23] Jones J. D. G. Dangl J. L. (2006). The plant immune system. Nature 444 (7117), 323–329. doi: 10.1038/nature05286 17108957

[B24] Kim H. Ahn Y. J. Lee H. Chung E. H. Segonzac C. Sohn K. H. (2023). Diversified host target families mediate convergently evolved effector recognition across plant species. Curr. Opin. Plant Biol. 74, 102398. doi: 10.1016/j.pbi.2023.102398 37295296

[B25] Kim M. C. Panstruga R. Elliott C. Müller J. Devoto A. Yoon H. W.. (2002). Calmodulin interacts with MLO protein to regulate defence against mildew in barley. Nature 416 (6879), 447–451. doi: 10.1038/416447a 11919636

[B26] Kloppe T. Whetten R. B. Kim S. B. Powell O. R. Lück S. Douchkov D.. (2023). Two pathogen loci determine Blumeria graminis f. sp. tritici virulence to wheat resistance gene Pm1a. New Phytol. 238 (4), 957-969. doi: 10.1111/nph.18809 36772855

[B27] Klymiuk V. Yaniv E. Huang L. Raats D. Fatiukha A. Chen S.. (2018). Cloning of the wheat Yr15 resistance gene sheds light on the plant tandem kinase-pseudokinase family. Nat. Commun. 9 (1), 3735. doi: 10.1038/s41467-018-06138-9 30282993 PMC6170490

[B28] Krattinger S. G. Kang J. Bräunlich S. Boni R. Chauhan H. Selter L. L.. (2019). Abscisic acid is a substrate of the ABC transporter encoded by the durable wheat disease resistance gene Lr34. New Phytol. 223 (2), 853–866. doi: 10.1111/nph.15815 30913300 PMC6618152

[B29] Krattinger S. G. Lagudah E. S. Spielmeyer W. Singh R. P. Huerta-Espino J. McFadden H.. (2009). A putative ABC transporter confers durable resistance to multiple fungal pathogens in wheat. Science 323 (5919), 1360–1363. doi: 10.1126/science.1166453 19229000

[B30] Kusch S. Panstruga R. (2017). mlo-based resistance: An apparently universal "Weapon" to defeat powdery mildew disease. Mol. Plant Microbe Interact. 30 (3), 179–189. doi: 10.1094/mpmi-12-16-0255-cr 28095124

[B31] Li M. Dong L. Li B. Wang Z. Xie J. Qiu D.. (2020). A CNL protein in wild emmer wheat confers powdery mildew resistance. New Phytol. 228 (3), 1027–1037. doi: 10.1111/nph.16761 32583535

[B32] Li S. Lin D. Zhang Y. Deng M. Chen Y. Lv B.. (2022). Genome-edited powdery mildew resistance in wheat without growth penalties. Nature 602 (7897), 455–460. doi: 10.1038/s41586-022-04395-9 35140403

[B33] Lu P. Guo L. Wang Z. Li B. Li J. Li Y.. (2020). A rare gain of function mutation in a wheat tandem kinase confers resistance to powdery mildew. Nat. Commun. 11 (1), 680–680. doi: 10.1038/s41467-020-14294-0 32015344 PMC6997164

[B37] Manser B. Koller T. Praz C. R. Roulin A. C. Zbinden H. Arora S.. (2021). Identification of specificity-defining amino acids of the wheat immune receptor Pm2 and powdery mildew effector AvrPm2. Plant J. 106 (4), 993–1007. doi: 10.1111/tpj.15214 33629439

[B38] Mapuranga J. Chang J. Yang W. (2022). Combating powdery mildew: Advances in molecular interactions between Blumeria graminis f. sp. tritici and wheat. Front. Plant Sci. 13. doi: 10.3389/fpls.2022.1102908 PMC9800938 36589137

[B39] Moore J. W. Herrera-Foessel S. Lan C. Schnippenkoetter W. Ayliffe M. Huerta-Espino J.. (2015). A recently evolved hexose transporter variant confers resistance to multiple pathogens in wheat. Nat. Genet. 47 (12), 1494–1498. doi: 10.1038/ng.3439 26551671

[B34] Müller M. C. Kunz L. Graf J. P. Schudel S. Keller B. (2021). Host adaptation through hybridization: Genome analysis of triticale powdery mildew reveals unique combination of lineage-specific effectors. Mol. Plant Microbe Interact. 34 (12), 1350-1357. doi: 10.1094/mpmi-05-21-0111-sc 34503345

[B35] Müller M. C. Kunz L. Schudel S. Lawson A. W. Kammerecker S. Isaksson J.. (2022). Ancient variation of the AvrPm17 gene in powdery mildew limits the effectiveness of the introgressed rye Pm17 resistance gene in wheat. Proc. Natl. Acad. Sci. U.S.A. 119 (30), e2108808119. doi: 10.1073/pnas.2108808119 35857869 PMC9335242

[B36] Müller M. C. Praz C. R. Sotiropoulos A. G. Menardo F. Kunz L. Schudel S.. (2018). A chromosome-scale genome assembly reveals a highly dynamic effector repertoire of wheat powdery mildew. New Phytol. 221 (4), 2176-2189. doi: 10.1111/nph.15529 PMC6587952 30388298

[B40] Najafi J. Palmgren M. (2022). Hexose transport reverts the growth penalty of mlo resistance. Trends Plant Sci. 27 (8), 739–741. doi: 10.1016/j.tplants.2022.04.003 35469738

[B41] Neubauer M. Serrano I. Rodibaugh N. Bhandari D. D. Bautor J. Parker J. E.. (2020). Arabidopsis EDR1 protein kinase regulates the association of EDS1 and PAD4 to inhibit cell death. Mol. Plant Microbe Interact. 33 (4), 693–703. doi: 10.1094/mpmi-12-19-0339-r 31876224 PMC8162682

[B42] Praz C. R. Bourras S. Zeng F. Sánchez-Martín J. Menardo F. Xue M.. (2017). AvrPm2 encodes an RNase-like avirulence effector which is conserved in the two different specialized forms of wheat and rye powdery mildew fungus. New Phytol. 213 (3), 1301–1314. doi: 10.1111/nph.14372 27935041 PMC5347869

[B43] Proels R. K. Hückelhoven R. (2014). Cell-wall invertases, key enzymes in the modulation of plant metabolism during defence responses. Mol. Plant Pathol. 15 (8), 858–864. doi: 10.1111/mpp.12139 24646208 PMC6638650

[B44] Rajaraman J. Douchkov D. Hensel G. Stefanato F. L. Gordon A. Ereful N.. (2016). An LRR/malectin receptor-like kinase mediates resistance to non-adapted and adapted powdery mildew fungi in barley and wheat. Front. Plant Sci. 7. doi: 10.3389/fpls.2016.01836 PMC5156707 28018377

[B45] Reynolds M. Foulkes J. Furbank R. Griffiths S. King J. Murchie E.. (2012). Achieving yield gains in wheat. Plant Cell Environ. 35 (10), 1799–1823. doi: 10.1111/j.1365-3040.2012.02588.x 22860982

[B46] Sánchez-Martín J. Keller B. (2021). NLR immune receptors and diverse types of non-NLR proteins control race-specific resistance in Triticeae. Curr. Opin. Plant Biol. 62, 102053. doi: 10.1016/j.pbi.2021.102053 34052730

[B48] Sanchez-Martin J. Steuernagel B. Ghosh S. Herren G. Hurni S. Adamski N.. (2016). Rapid gene isolation in barley and wheat by mutant chromosome sequencing. Genome Biol. 17 (1), 221. doi: 10.1186/s13059-016-1082-1 27795210 PMC5087116

[B47] Sánchez-Martín J. Widrig V. Herren G. Wicker T. Zbinden H. Gronnier J.. (2021). Wheat Pm4 resistance to powdery mildew is controlled by alternative splice variants encoding chimeric proteins. Nat. Plants 7 (3), 327–341. doi: 10.1038/s41477-021-00869-2 33707738 PMC7610370

[B49] Savary S. Willocquet L. Pethybridge S. J. Esker P. McRoberts N. Nelson A. (2019). The global burden of pathogens and pests on major food crops. Nat. Ecol. Evol. 3 (3), 430–439. doi: 10.1038/s41559-018-0793-y 30718852

[B50] Schwarzbach E. (1979). Response to selection for virulence against the ml-o based resistance in barley, not fitting the gene-for-gene hypothesis. BARLEY Genet. Newslett. 9, 85–89.

[B52] Singh S. P. Hurni S. Ruinelli M. Brunner S. Sanchez-Martin J. Krukowski P.. (2018). Evolutionary divergence of the rye *Pm17* and *Pm8* resistance genes reveals ancient diversity. Plant Mol. Biol. 98 (3), 249–260. doi: 10.1007/s11103-018-0780-3 30244408

[B51] Singh R. P. Singh P. K. Rutkoski J. Hodson D. P. He X. Jorgensen L. N.. (2016). Disease impact on wheat yield potential and prospects of genetic control. Annu. Rev. Phytopathol. 54, 303–322. doi: 10.1146/annurev-phyto-080615-095835 27296137

[B53] Sotiropoulos A. G. Arango-Isaza E. Ban T. Barbieri C. Bourras S. Cowger C.. (2022). Global genomic analyses of wheat powdery mildew reveal association of pathogen spread with historical human migration and trade. Nat. Commun. 13 (1), 4315. doi: 10.1038/s41467-022-31975-0 35882860 PMC9315327

[B54] Srichumpa P. Brunner S. Keller B. Yahiaoui N. (2005). Allelic series of four powdery mildew resistance genes at the Pm3 locus in hexaploid bread wheat. Plant Physiol. 139 (2), 885–895. doi: 10.1104/pp.105.062406 16183849 PMC1256003

[B55] Tang D. Wang G. Zhou J.-M. (2017). Receptor kinases in plant-pathogen interactions: more than pattern recognition. Plant Cell 29 (4), 618–637. doi: 10.1105/tpc.16.00891 28302675 PMC5435430

[B57] Wang Z. Cheng J. Fan A. Zhao J. Yu Z. Li Y.. (2018). LecRK-V, an L-type lectin receptor kinase in Haynaldia villosa, plays positive role in resistance to wheat powdery mildew. Plant Biotechnol. J. 16 (1), 50–62. doi: 10.1111/pbi.12748 28436098 PMC5811777

[B56] Wang Y. Cheng X. Shan Q. Zhang Y. Liu J. Gao C.. (2014). Simultaneous editing of three homoeoalleles in hexaploid bread wheat confers heritable resistance to powdery mildew. Nat. Biotech. advance Online Publ. 32 (9), 947-951. doi: 10.1038/nbt.2969 25038773

[B58] Xia T. Yang Y. Zheng H. Han X. Jin H. Xiong Z.. (2021). Efficient expression and function of a receptor-like kinase in wheat powdery mildew defence require an intron-located MYB binding site. Plant Biotechnol. J. 19 (5), 897–909. doi: 10.1111/pbi.13512 33225586 PMC8131041

[B59] Xie J. Guo G. Wang Y. Hu T. Wang L. Li J.. (2020). A rare single nucleotide variant in Pm5e confers powdery mildew resistance in common wheat. New Phytol. 228 (3), 1011-1026. doi: 10.1111/nph.16762 32569398

[B60] Xing L. Hu P. Liu J. Witek K. Zhou S. Xu J.. (2018). Pm21 from haynaldia villosa encodes a CC-NBS-LRR protein conferring powdery mildew resistance in wheat. Mol. Plant 11 (6), 874–878. doi: 10.1016/j.molp.2018.02.013 29567451

[B61] Yahiaoui N. Srichumpa P. Dudler R. Keller B. (2004). Genome analysis at different ploidy levels allows cloning of the powdery mildew resistance gene *Pm3b* from hexaploid wheat. Plant J. 37 (4), 528–538. doi: 10.1046/j.1365-313X.2003.01977.x 14756761

[B62] Yuan M. Ngou B. P. M. Ding P. Xin X.-F. (2021). PTI-ETI crosstalk: an integrative view of plant immunity. Curr. Opin. Plant Biol. 62, 102030. doi: 10.1016/j.pbi.2021.102030 33684883

[B63] Zhang Y. Bai Y. Wu G. Zou S. Chen Y. Gao C.. (2017). Simultaneous modification of three homoeologs of TaEDR1 by genome editing enhances powdery mildew resistance in wheat. Plant J. 91 (4), 714–724. doi: 10.1111/tpj.13599 28502081

[B64] Zhao C. Nie H. Shen Q. Zhang S. Lukowitz W. Tang D. (2014). EDR1 physically interacts with MKK4/MKK5 and negatively regulates a MAP kinase cascade to modulate plant innate immunity. PloS Genet. 10 (5), e1004389. doi: 10.1371/journal.pgen.1004389 24830651 PMC4022593

[B65] Zhu S. Liu C. Gong S. Chen Z. Chen R. Liu T.. (2023). Orthologous genes Pm12 and Pm21 from two wild relatives of wheat show evolutionary conservation but divergent powdery mildew resistance. Plant Commun. 4 (2), 100472. doi: 10.1016/j.xplc.2022.100472 36352792 PMC10030366

[B66] Zou S. Shi W. Ji J. Wang H. Tang Y. Yu D.. (2022). Diversity and similarity of wheat powdery mildew resistance among three allelic functional genes at the Pm60 locus. Plant J. 110 (6), 1781–1790. doi: 10.1111/tpj.15771 35411560

[B67] Zou S. H. Wang H. Li Y. W. Kong Z. S. Tang D. Z. (2018). The NB-LRR gene Pm60 confers powdery mildew resistance in wheat. New Phytol. 218 (1), 298–309. doi: 10.1111/nph.14964 29281751

