# Supplementary material for: Wheat powdery mildew resistance: from gene identification to immunity deployment
Source: Front Plant Sci. 2023 Sep 18;14:1269498. doi: 10.3389/fpls.2023.1269498 (PMC10544919; doi:10.3389/fpls.2023.1269498)
Supplement: Supplementary file 1 [file Table_1.docx]

Table S1 List of reported wheat powdery mildew (*Pm*) resistance alleles in bread wheat and its relatives.

| *Pm* alleles | Chromosomal localization |
| --- | --- |
| *Pm3a-3j* ([Huang et al., 2004](#_ENREF_21)) | 1AS |
| *Pm3k* ([Yahiaoui et al., 2009](#_ENREF_77)) | 1AS |
| *Pm17* ([Hsam and Zeller, 1997](#_ENREF_18)) | T1AL.1RS |
| *Pm25* ([Shi et al., 1998](#_ENREF_58)) | 1A |
| *PmCn17* ([Ren et al., 2009](#_ENREF_54)) | T1BL.1RS |
| *Pm8* ([Hsam and Zeller, 1997](#_ENREF_18)) | T1BL.1RS |
| *Pm32* ([Hsam et al., 2003](#_ENREF_17)) | T1BL.1SS |
| *Pm39* ([Lillemo et al., 2008](#_ENREF_31)) | 1BL |
| *Pm28* ([Peusha et al., 2000](#_ENREF_49)) | 1B |
| *Pm24* ([Huang and Röder, 2011](#_ENREF_22)) | 1DS |
| *Mlbhl* ([Xue et al., 2012](#_ENREF_76)) | 1DS |
| *Pm10* ([Tosa et al., 1987](#_ENREF_66)) | 1D |
| *Pm57* ([Liu et al., 2017](#_ENREF_32)) | T2BS.2BL-2Ss#1L |
| *Pm4a* ([Ma et al., 2004](#_ENREF_39)) | 2AL |
| *Pm4b* ([T et al., 1979](#_ENREF_63)) | 2AL |
| *Pm4c/Pm23* ([Hao et al., 2008](#_ENREF_11)) | 2AL |
| *Pm4d* ([Schmolke et al., 2012](#_ENREF_56)) | 2AL |
| *PmLK906* ([Niu et al., 2010](#_ENREF_47)) | 2AL |
| *PmHNK54* ([Xu et al., 2011](#_ENREF_73)) | 2AL |
| *PmPS5A* ([Zhu et al., 2005](#_ENREF_86)) | 2AL |
| *PmYm66* ([Hu et al., 2008](#_ENREF_19)) | 2AL |
| *PmX* ([Fu et al., 2013](#_ENREF_10))(recessive) | 2AL |
| *Pm351817* ([Xu et al., 2023](#_ENREF_75)) | 2AL |
| *Pm65* ([Li et al., 2019](#_ENREF_28)) | 2AL |
| *PmE* ([Zhou et al., 2005](#_ENREF_84)) | 2AL |
| *MlTd1055* ([Ahmadi and Moore, 2007](#_ENREF_1)) | 2A |
| *Ml5323* ([Piarulli et al., 2012](#_ENREF_50)) | 2BS |
| *MlWE74* ([Zhu et al., 2022](#_ENREF_85)) | 2BS |
| *MlIW170* ([Liu et al., 2012](#_ENREF_34)) | 2BS |
| *Pm26* ([Rong et al., 2000](#_ENREF_55)) (recessive) | 2BS |
| *Pm42* ([Hua et al., 2009](#_ENREF_20)) (recessive) | 2BS |
| *Pm6* ([Qin et al., 2011](#_ENREF_51)) | 2BL |
| *pmYN99102* ([Mu et al., 2022](#_ENREF_46)) | 2BL |
| *PmCG15-009* ([Zhang et al., 2023](#_ENREF_82)) | 2BL |
| *Pm33* ([Zhu et al., 2005](#_ENREF_86)) | 2BL |
| *Pm50* ([Mohler et al., 2013](#_ENREF_44)) | 2BL |
| *MlLX99* ([Zhao et al., 2013](#_ENREF_83)) | 2BL |
| *PmJM22* ([Yin et al., 2009](#_ENREF_79)) | 2BL |
| *MlZec1* ([Mohler et al., 2005](#_ENREF_45)) | 2BL |
| *MlAB10* ([Maxwell et al., 2010](#_ENREF_41)) | 2BL |
| *MlIW39 (*[*Qiu et al., 2021*](#_ENREF_52)*)* | 2B |
| *PmY39* ([Zhu et al., 2006](#_ENREF_87)) | 2U(2B) |
| *Pm58* ([Wiersma et al., 2017](#_ENREF_68)) | 2DS |
| *Pm43* ([He et al., 2009](#_ENREF_13)) | 2DL |
| *Pm13* ([Cenci et al., 1999](#_ENREF_5)) | T3BL.3BS-3S^l^S |
| *QPm.caas-3BS* ([Jia et al., 2018](#_ENREF_27)) | 3BS |
| *PmHNK* ([Xu et al., 2010](#_ENREF_74)) | 3BL |
| *Pm41* ([Li et al., 2009](#_ENREF_29)) | 3BL |
| *PmPBDH* ([Liang et al., 2022](#_ENREF_30)) | 4AL |
| *Pm7* ([Friebe et al., 1996](#_ENREF_9)) | T4BS.4BL-2RL |
| *Pm46* ([Hiebert et al., 2010](#_ENREF_14)) | 4DL |
| *Pm2026* ([Xu et al., 2008](#_ENREF_71)) (recessive) | 5AL |
| *Pm16* ([Chen et al., 2005](#_ENREF_6)) | 5BS |
| *Pm30* ([Liu et al., 2002](#_ENREF_33)) | 5BS |
| *Pm36* ([Blanco et al., 2008](#_ENREF_3)) | 5BL |
| *Ml3D32* ([Zhang et al., 2010](#_ENREF_81)) | 5BL |
| *Pm34* ([Miranda et al., 2006](#_ENREF_43)) | 5DL |
| *Pm35* ([Miranda et al., 2007](#_ENREF_42)) | 5DL |
| *Pm2* ([Lutz et al., 1995](#_ENREF_36)) | 5DS |
| *Pm2b* ([Ma et al., 2015](#_ENREF_38)) | 5DS |
| *Pm2c* ([Xu et al., 2015](#_ENREF_72)) | 5DS |
| *Pm21* ([Cao et al., 2011](#_ENREF_4)) | T6VS·6AL |
| *Pm11* ([Tosa et al., 1988](#_ENREF_65)) | 6BS |
| *Pm12* ([Song et al., 2007](#_ENREF_61)) | T6BS-6SS.6SL |
| *Pm20* ([Friebe et al., 1994](#_ENREF_8)) | T6BS.6RL |
| *Pm27* ([Järve K et al., 2000](#_ENREF_25)) | 6B |
| *Pm14* ([Tosa and Sakai, 1990](#_ENREF_64)) | 6B |
| *Pm54* ([Hao et al., 2015](#_ENREF_12)) | 6BL |
| *Pm45* ([Ma et al., 2011](#_ENREF_37)) | 6DS |
| *Pm1a* ([Hsam et al., 1998](#_ENREF_15)) | 7AL |
| *Pm1b* ([Hsam et al., 1998](#_ENREF_15)) | 7AL |
| *Pm1c-Pm18* ([Hsam et al., 1998](#_ENREF_15)) | 7AL |
| *Pm1d* ([Hsam et al., 1998](#_ENREF_15)) | 7AL |
| *Pm1e-Pm22* ([Singrün et al., 2003](#_ENREF_59)) | 7AL |
| *Pm9* ([Schneider et al., 1991](#_ENREF_57)) | 7AL |
| *PmU* ([Qiu et al., 2005](#_ENREF_53)) | 7AL |
| *MlIW172* ([Wu et al., 2022](#_ENREF_69)) | 7AL |
| *Pm60* ([Zou et al., 2018](#_ENREF_88)) | 7AL |
| *Pm37* ([Perugini et al., 2008](#_ENREF_48)) | 7AL |
| *PmG16* ([Ben-David et al., 2010](#_ENREF_2)) | 7AL |
| *MlIw72* ([Ji et al., 2008](#_ENREF_26)) | 7AL |
| *PmTb7A.1* ([Ma et al., 2015](#_ENREF_38)) | 7AL |
| *Mlm203* ([Yao et al., 2007](#_ENREF_78)) | 7AL |
| *Mlm80* ([Yao et al., 2007](#_ENREF_78)) | 7AL |
| *MlAG12* ([Maxwell et al., 2009](#_ENREF_40)) | 7AL |
| *mlRd30* ([Singrün et al., 2004](#_ENREF_60)) (recessive) | 7AL |
| *Pm40* ([Luo et al., 2009](#_ENREF_35)) | 7BS |
| *Pm47* ([Xiao et al., 2013](#_ENREF_70)) | 7BS |
| *Pm5a* ([Hsam et al., 2001](#_ENREF_16)) (recessive) | 7BL |
| *pmHYM* ([Wang et al., 2022](#_ENREF_67)) | 7BL |
| *Pm5b* ([Hsam et al., 2001](#_ENREF_16)) (recessive) | 7BL |
| *Pm5c* ([Hsam et al., 2001](#_ENREF_16)) (recessive) | 7BL |
| *Pm5d* ([Hsam et al., 2001](#_ENREF_16)) (recessive) | 7BL |
| *Pm5e* ([Huang et al., 2003](#_ENREF_23)) (recessive) | 7BL |
| *mljy* ([Huang et al., 2002](#_ENREF_24)) (recessive) | 7BL |
| *mlsy* ([Huang et al., 2002](#_ENREF_24)) (recessive) | 7BL |
| *Mlxbd* ([Fei et al., 2009](#_ENREF_7)) (recessive) | 7BL |
| *Pm15* ([Tosa and Sakai, 1990](#_ENREF_64)) | 7DS |
| *Pm38* ([Spielmeyer et al., 2005](#_ENREF_62)) | 7DS |
| *Pm29* ([Zeller et al., 2002](#_ENREF_80)) | 7DL |
| *Pm19* ([Lutz et al., 1995](#_ENREF_36)) | 7D |

Reference

Ahmadi, H., and Moore, K. (2007). Inheritance and chromosomal location of powdery mildew resistance gene in wild wheat *Triticum turgidum* Var. *dicoccoides*. *J Plant Pathol* 6**,** 164-168.

Ben-David, R., Xie, W., Peleg, Z., Saranga, Y., Dinoor, A., and Fahima, T. (2010). Identification and mapping of *PmG16*, a powdery mildew resistance gene derived from wild emmer wheat. *Theoretical and Applied Genetics* 121(3)**,** 499-510. doi: 10.1007/s00122-010-1326-5.

Blanco, A., Gadaleta, A., Cenci, A., Carluccio, A.V., Abdelbacki, A.M.M., and Simeone, R. (2008). Molecular mapping of the novel powdery mildew resistance gene *Pm36* introgressed from *Triticum turgidum* var. *dicoccoides* in durum wheat. *Theoretical and Applied Genetics* 117(1)**,** 135-142. doi: 10.1007/s00122-008-0760-0.

Cao, A., Xing, L., Wang, X., Yang, X., Wang, W., Sun, Y., et al. (2011). Serine/threonine kinase gene *Stpk-V*, a key member of powdery mildew resistance gene *Pm21*, confers powdery mildew resistance in wheat. *Proceedings of the National Academy of Sciences, USA* 108(19)**,** 7727-7732.

Cenci, A., D’Ovidio, R., Tanzarella, O.A., Ceoloni, C., and Porceddu, E. (1999). Identification of molecular markers linked to *Pm13*, an *Aegilops longissima* gene conferring resistance to powdery mildew in wheat. *Theoretical and Applied Genetics* 98(3-4)**,** 448-454. doi: 10.1007/s001220051090.

Chen, X.M., Luo, Y.H., Xia, X.C., Xia, L.Q., Chen, X., Ren, Z.L., et al. (2005). Chromosomal location of powdery mildew resistance gene *Pm16* in wheat using SSR marker analysis. *Plant Breeding* 124(3)**,** 225-228. doi: 10.1111/j.1439-0523.2005.01094.x.

Fei, X., Zhai, W., Dian, X., Zhou, Y., and JI, W. (2009). Microsatellite mapping of powdery mildew resistance gene in wheat landrace Xiaobaidong. *Acta Agron Sin* 35(10)**,** 1806-1811. doi: 10.3724/sp.j.1006.2009.01806.

Friebe, B., Heun, M., Tuleen, N., Zeller, F.J., and Gill, B.S. (1994). Cytogenetically monitored transfer of powdery mildew resistance from rye into wheat. *Crop Sci.* 34(3)**,** 621-625. doi: 10.2135/cropsci1994.0011183X003400030003x.

Friebe, B., Jiang, J., Raupp, W.J., McIntosh, R.A., and Gill, B.S. (1996). Characterization of wheat-alien translocations conferring resistance to diseases and pests: current status. *Euphytica* 91**,** 59-87. doi: 10.1007/BF00035277.

Fu, B., Chen, Y., Li, N., Ma, H., Kong, Z., Zhang, L., et al. (2013). *PmX*: a recessive powdery mildew resistance gene at the *Pm4* locus identified in wheat landrace Xiaohongpi. *Theoretical and Applied Genetics* 126(4)**,** 913-921. doi: 10.1007/s00122-012-2025-1.

Hao, Y., Liu, A., Wang, Y., Feng, D., Gao, J., Li, X., et al. (2008). *Pm23*: a new allele of *Pm4* located on chromosome 2AL in wheat. *Theoretical and Applied Genetics* 117(8)**,** 1205-1212. doi: 10.1007/s00122-008-0827-y.

Hao, Y., Parks, R., Cowger, C., Chen, Z., Wang, Y., Bland, D., et al. (2015). Molecular characterization of a new powdery mildew resistance gene *Pm54* in soft red winter wheat. *Theoretical and Applied Genetics* 128(3)**,** 465-476. doi: 10.1007/s00122-014-2445-1.

He, R., Chang, Z., Yang, Z., Yuan, Z., Zhan, H., Zhang, X., et al. (2009). Inheritance and mapping of powdery mildew resistance gene *Pm43* introgressed from *Thinopyrum intermedium* into wheat. *Theoretical and Applied Genetics* 118(6)**,** 1173-1180. doi: 10.1007/s00122-009-0971-z.

Hiebert, C., Thomas, J., McCallum, B., Humphreys, D.G., DePauw, R., Hayden, M., et al. (2010). An introgression on wheat chromosome 4DL in RL6077 (Thatcher*6/PI 250413) confers adult plant resistance to stripe rust and leaf rust (*Lr67*). *Theoretical and Applied Genetics* 121(6)**,** 1083-1091. doi: 10.1007/s00122-010-1373-y.

Hsam, S.L.K., Huang, X.Q., Ernst, F., Hartl, L., and Zeller, F.J. (1998). Chromosomal location of genes for resistance to powdery mildew in common wheat (*Triticum aestivum* L. em Thell.). *Theoretical and Applied Genetics* 96(8)**,** 1129-1134. doi: 10.1007/s001220050848.

Hsam, S.L.K., Huang, X.Q., and Zeller, F.J. (2001). Chromosomal location of genes for resistance to powdery mildew in common wheat (*Triticum aestivum* L. em Thell.) 6. alleles at the *Pm5* locus. *Theor Appl Genet* 102(1)**,** 127-133. doi: 10.1007/s001220051627.

Hsam, S.L.K., Lapochkina, I.F., and Zeller, F.J. (2003). Chromosomal location of genes for resistance to powdery mildew in common wheat (*Triticum aestivum* L. em Thell.). *Euphytica* 133(3)**,** 367-370. doi: 10.1023/A:1025738513638.

Hsam, S.L.K., and Zeller, F.J. (1997). Evidence of allelism between genes *Pm8* and *Pm17* and chromosomal location of powdery mildew and leaf rust resistance genes in the common wheat cultivar ‘Amigo'. *Plant Breeding* 116(2)**,** 119-122. doi: 10.1111/j.1439-0523.1997.tb02164.x.

Hu, T., Li, H., Liu, Z., Xie, C., Zhou, Y., Duan, X., et al. (2008). Identification and molecular mapping of the powdery mildew resistance gene in wheat cultivar Yumai 66. *Acta Agron Sin* 34**,** 545-550.

Hua, W., Liu, Z., Zhu, J., Xie, C., Yang, T., Zhou, Y., et al. (2009). Identification and genetic mapping of *Pm42*, a new recessive wheat powdery mildew resistance gene derived from wild emmer (*Triticum turgidum* var. *dicoccoides*). *Theoretical and Applied Genetics* 119(2)**,** 223-230. doi: 10.1007/s00122-009-1031-4.

Huang, X.-Q., Hsam, S.L.K., Mohler, V., Röder, M.S., and Zeller, F.J. (2004). Genetic mapping of three alleles at the *Pm3* locus conferring powdery mildew resistance in common wheat (*Triticum aestivum* L.). *Genome* 47(6)**,** 1130-1136. doi: 10.1139/g04-079.

Huang, X.-Q., and Röder, M. (2011). High-density genetic and physical bin mapping of wheat chromosome 1D reveals that the powdery mildew resistance gene *Pm24* is located in a highly recombinogenic region. *Genetica* 139(9)**,** 1179-1187. doi: 10.1007/s10709-011-9620-y.

Huang, X., Wang, L., Xu, M., and Röder, M. (2003). Microsatellite mapping of the powdery mildew resistance gene *Pm5e* in common wheat (*Triticum aestivum* L.). *Theoretical and Applied Genetics* 106(5)**,** 858-865. doi: 10.1007/s00122-002-1146-3.

Huang, X.Q., Hsam, S.L.K., and Zeller, F.J. (2002). Chromosomal location of genes for resistance to powdery mildew in Chinese wheat lines Jieyan 94-1-1 and Siyan 94-1-2. *Hereditas* 136(3)**,** 212-218. doi: 10.1034/j.1601-5223.2002.t01-1-1360306.x.

Järve K, Peusha HO, Tsymbalova J, Tamm S, Devos KM, and TM., E. (2000). Chromosomal location of a *Triticum timopheevii*-derived powdery mildew resistance gene transferred to common wheat. *Genome* 43(2)**,** 377-381.

Ji, X., Xie, C., Ni, Z., Yang, T., Nevo, E., Fahima, T., et al. (2008). Identification and genetic mapping of a powdery mildew resistance gene in wild emmer (*Triticum dicoccoides*) accession IW72 from Israel. *Euphytica* 159(3)**,** 385-390. doi: 10.1007/s10681-007-9540-1.

Jia, A., Ren, Y., Gao, F., Yin, G., Liu, J., Guo, L., et al. (2018). Mapping and validation of a new QTL for adult-plant resistance to powdery mildew in Chinese elite bread wheat line Zhou8425B. *Theoretical and Applied Genetics*. doi: 10.1007/s00122-018-3058-x.

Li, G., Cowger, C., Wang, X., Carver, B.F., and Xu, X. (2019). Characterization of Pm65, a new powdery mildew resistance gene on chromosome 2AL of a facultative wheat cultivar. *Theoretical and Applied Genetics*. doi: 10.1007/s00122-019-03377-2.

Li, G., Fang, T., Zhang, H., Xie, C., Li, H., Yang, T., et al. (2009). Molecular identification of a new powdery mildew resistance gene *Pm41* on chromosome 3BL derived from wild emmer (*Triticum turgidum* var. *dicoccoides*). *Theoretical and Applied Genetics* 119(3)**,** 531-539. doi: 10.1007/s00122-009-1061-y.

Liang, X., Xu, H., Zhu, S., Zheng, Y., Zhong, W., Li, H., et al. (2022). Genetically Dissecting the Novel Powdery Mildew Resistance Gene in the Wheat Breeding Line PBDH1607. *Plant Dis*. doi: 10.1094/pdis-12-21-2771-re.

Lillemo, M., Asalf, B., Singh, R.P., Huerta-Espino, J., Chen, X.M., He, Z.H., et al. (2008). The adult plant rust resistance loci *Lr34/Yr18* and *Lr46/Yr29* are important determinants of partial resistance to powdery mildew in bread wheat line Saar. *Theoretical and Applied Genetics* 116(8)**,** 1155-1166. doi: 10.1007/s00122-008-0743-1.

Liu, W., Koo, D.-H., Xia, Q., Li, C., Bai, F., Song, Y., et al. (2017). Homoeologous recombination-based transfer and molecular cytogenetic mapping of powdery mildew-resistant gene Pm57 from Aegilops searsii into wheat. *Theoretical and Applied Genetics* 130(4)**,** 841-848. doi: 10.1007/s00122-017-2855-y.

Liu, Z., Sun, Q., Ni, Z., Nevo, E., and Yang, T. (2002). Molecular characterization of a novel powdery mildew resistance gene *Pm30* in wheat originating from wild emmer. *Euphytica* 123(1)**,** 21-29. doi: 10.1023/A:1014471113511.

Liu, Z., Zhu, J., Cui, Y., Liang, Y., Wu, H., Song, W., et al. (2012). Identification and comparative mapping of a powdery mildew resistance gene derived from wild emmer (*Triticum turgidum* var. *dicoccoides*) on chromosome 2BS. *Theoretical and Applied Genetics* 124(6)**,** 1041-1049. doi: 10.1007/s00122-011-1767-5.

Luo, P.G., Luo, H.Y., Chang, Z.J., Zhang, H.Y., Zhang, M., and Ren, Z.L. (2009). Characterization and chromosomal location of *Pm40* in common wheat: a new gene for resistance to powdery mildew derived from *Elytrigia intermedium*. *Theoretical and Applied Genetics* 118(6)**,** 1059-1064. doi: 10.1007/s00122-009-0962-0.

Lutz, J., Hsam, S.L.K., Limpert, E., and Zeller, F.J. (1995). Chromosomal location of powdery mildew resistance genes in *Triticum aestivum* L. (common wheat). 2. genes *Pm2* and *Pm19* from *Aegilops squarrosa* L. *Heredity* 74(2)**,** 152-156.

Ma, H., Kong, Z., Fu, B., Li, N., Zhang, L., Jia, H., et al. (2011). Identification and mapping of a new powdery mildew resistance gene on chromosome 6D of common wheat. *Theoretical and Applied Genetics* 123(7)**,** 1099-1106. doi: 10.1007/s00122-011-1651-3.

Ma, P., Xu, H., Xu, Y., Li, L., Qie, Y., Luo, Q., et al. (2015). Molecular mapping of a new powdery mildew resistance gene *Pm2b* in Chinese breeding line KM2939. *Theoretical and Applied Genetics* 128(4)**,** 613-622. doi: 10.1007/s00122-015-2457-5.

Ma, Z.Q., Wei, J.B., and Cheng, S.H. (2004). PCR-based markers for the powdery mildew resistance gene *Pm4a* in wheat. *Theor Appl Genet* 109(1)**,** 140-145. doi: 10.1007/s00122-004-1605-0.

Maxwell, J., Lyerly, J., Cowger, C., Marshall, D., Brown-Guedira, G., and Murphy, J.P. (2009). *MlAG12*: a *Triticum timopheevii*-derived powdery mildew resistance gene in common wheat on chromosome 7AL. *Theoretical and Applied Genetics* 119(8)**,** 1489-1495. doi: 10.1007/s00122-009-1150-y.

Maxwell, J.J., Lyerly, J.H., Srnic, G., Parks, R., Cowger, C., Marshall, D., et al. (2010). *MlAB10* : a *Triticum turgidum* Subsp. *dicoccoides* derived powdery mildew resistance gene identified in common wheat. *Crop Sci.* 50(6)**,** 2261-2267. doi: 10.2135/cropsci2010.04.0195.

Miranda, L.M., Murphy, J.P., Marshall, D., Cowger, C., and Leath, S. (2007). Chromosomal location of *Pm35*, a novel *Aegilops tauschii* derived powdery mildew resistance gene introgressed into common wheat (*Triticum aestivum* L.). *Theoretical and Applied Genetics* 114(8)**,** 1451-1456. doi: 10.1007/s00122-007-0530-4.

Miranda, L.M., Murphy, J.P., Marshall, D., and Leath, S. (2006). *Pm34:* a new powdery mildew resistance gene transferred from *Aegilops tauschii* Coss. to common wheat (*Triticum aestivum* L.). *Theoretical and Applied Genetics* 113(8)**,** 1497-1504. doi: 10.1007/s00122-006-0397-9.

Mohler, V., Bauer, C., Schweizer, G., Kempf, H., and Hartl, L. (2013). *Pm50*: a new powdery mildew resistance gene in common wheat derived from cultivated emmer. *J Appl Genet* 54(3)**,** 259-263. doi: 10.1007/s13353-013-0158-9.

Mohler, V., Zeller, F., Wenzel, G., and Hsam, S.K. (2005). Chromosomal location of genes for resistance to powdery mildew in common wheat (*Triticum aestivum* L. em Thell.). *Euphytica* 142(1-2)**,** 161-167. doi: 10.1007/s10681-005-1251-x.

Mu, Y., Gong, W., Qie, Y., Liu, X., Li, L., Sun, N., et al. (2022). Identification of the powdery mildew resistance gene in wheat breeding line Yannong 99102-06188 via bulked segregant exome capture sequencing. *Front Plant Sci* 13**,** 1005627. doi: 10.3389/fpls.2022.1005627.

Niu, J.-s., Jia, H.-y., Yin, J., Wang, B.-q., Ma, Z.-q., and Shen, T.-m. (2010). Development of an STS marker linked to powdery mildew resistance genes *PmLK906* and *Pm4a* by gene chip hybridization. *Agr Sci China* 9(3)**,** 331-336. doi: 10.1016/s1671-2927(09)60101-2.

Perugini, L.D., Murphy, J.P., Marshall, D., and Brown-Guedira, G. (2008). *Pm37*, a new broadly effective powdery mildew resistance gene from *Triticum timopheevii*. *Theoretical and Applied Genetics* 116(3)**,** 417-425. doi: 10.1007/s00122-007-0679-x.

Peusha, H., Enno, T., and Priilinn, O. (2000). Chromosomal location of powdery mildew resistance genes and cytogenetic analysis of meiosis in common wheat cultivar Meri. *Hereditas* 132(1)**,** 29-34. doi: 10.1111/j.1601-5223.2000.00029.x.

Piarulli, L., Gadaleta, A., Mangini, G., Signorile, M.A., Pasquini, M., Blanco, A., et al. (2012). Molecular identification of a new powdery mildew resistance gene on chromosome 2BS from *Triticum turgidum* ssp. *dicoccum*. *Plant Sci* 196(0)**,** 101-106. doi: 10.1016/j.plantsci.2012.07.015.

Qin, B., Cao, A., Wang, H., Chen, T., You, F., Liu, Y., et al. (2011). Collinearity-based marker mining for the fine mapping of *Pm6*, a powdery mildew resistance gene in wheat. *Theoretical and Applied Genetics* 123(2)**,** 207-218. doi: 10.1007/s00122-011-1577-9.

Qiu, L., Liu, N., Wang, H., Shi, X., Li, F., Zhang, Q., et al. (2021). Fine mapping of a powdery mildew resistance gene MlIW39 derived from wild emmer wheat (Triticum turgidum ssp. dicoccoides). *Theor Appl Genet* 134(8)**,** 2469-2479. doi: 10.1007/s00122-021-03836-9.

Qiu, Y.C., Zhou, R.H., Kong, X.Y., Zhang, S.S., and Jia, J.Z. (2005). Microsatellite mapping of a *Triticum urartu* Tum. derived powdery mildew resistance gene transferred to common wheat (*Triticum aestivum* L.). *Theor Appl Genet* 111(8)**,** 1524-1531. doi: 10.1007/s00122-005-0081-5.

Ren, T.-H., Yang, Z.-J., Yan, B.-J., Zhang, H.-Q., Fu, S.-L., and Ren, Z.-L. (2009). Development and characterization of a new 1BL.1RS translocation line with resistance to stripe rust and powdery mildew of wheat. *Euphytica* 169(2)**,** 207-213. doi: 10.1007/s10681-009-9924-5.

Rong, J.K., Millet, E., Manisterski, J., and Feldman, M. (2000). A new powdery mildew resistance gene: Introgression from wild emmer into common wheat and RFLP-based mapping. *Euphytica* 115(2)**,** 121-126. doi: 10.1023/A:1003950431049.

Schmolke, M., Mohler, V., Hartl, L., Zeller, F., and Hsam, S.K. (2012). A new powdery mildew resistance allele at the *Pm4* wheat locus transferred from einkorn (*Triticum monococcum*). *Mol Breed* 29(2)**,** 449-456. doi: 10.1007/s11032-011-9561-2.

Schneider, D.M., Heun, M., and Fischbeck, G. (1991). Inheritance of the powdery mildew resistance gene *Pm9* in relation to *Pm1* and *Pm2* of wheat. *Plant Breeding* 107(2)**,** 161-164. doi: 10.1111/j.1439-0523.1991.tb00545.x.

Shi, A.N., Leath, S., and Murphy, J.P. (1998). A major gene for powdery mildew resistance transferred to common wheat from wild einkorn wheat. *Phytopathology* 88(2)**,** 144-147. doi: 10.1094/phyto.1998.88.2.144.

Singrün, C., Hsam, S.L.K., Hartl, L., Zeller, F.J., and Mohler, V. (2003). Powdery mildew resistance gene *Pm22* in cultivar Virest is a member of the complex *Pm1* locus in common wheat (*Triticum aestivum* L. em Thell.). *Theor Appl Genet* 106(8)**,** 1420-1424. doi: 10.1007/s00122-002-1187-7.

Singrün, C., Hsam, S.L.K., Zeller, F.J., Wenzel, G., and Mohler, V. (2004). Localization of a novel recessive powdery mildew resistance gene from common wheat line RD30 in the terminal region of chromosome 7AL. *Theor Appl Genet* 109(1)**,** 210-214. doi: 10.1007/s00122-004-1619-7.

Song, W., Xie, H., Liu, Q., Xie, C., Ni, Z., Yang, T., et al. (2007). Molecular identification of *Pm12*-carrying introgression lines in wheat using genomic and EST-SSR markers. *Euphytica* 158(1-2)**,** 95-102. doi: 10.1007/s10681-007-9432-4.

Spielmeyer, W., McIntosh, R.A., Kolmer, J., and Lagudah, E.S. (2005). Powdery mildew resistance and *Lr34/Yr18* genes for durable resistance to leaf and stripe rust cosegregate at a locus on the short arm of chromosome 7D of wheat. *Theor Appl Genet* 111(4)**,** 731-735. doi: 10.1007/s00122-005-2058-9.

T, T.T., A, M.R., and A, B.F.G. (1979). Cytogenetic studies in wheat IX monosomic analysis telocentric mapping and linkage relationships of genes *Sr21, Pm4* and *Mle*. *Aust J Biol Sci.* 32**,** 115-125.

Tosa, Y., and Sakai, K. (1990). The genetics of resistance of hexaploid wheat to the wheatgrass powdery mildew fungus. *Genome* 33(2)**,** 225-230. doi: 10.1139/g90-035.

Tosa, Y., Tokunaga, H., and Ogura, H. (1988). Identification of a gene for resistance to wheatgrass powdery mildew fungus in common wheat cultivar Chinese Spring. *Genome* 30**,** 612-614.

Tosa, Y., Tsujimoto, H., and Ogura, H. (1987). A gene involved in the resistance of wheat to wheatgrass powdery mildew fungus. *Genome* 29(6)**,** 850-852. doi: 10.1139/g87-145.

Wang, J., Li, Y., Xu, F., Xu, H., Han, Z., Liu, L., et al. (2022). Candidate powdery mildew resistance gene in wheat landrace cultivar Hongyoumai discovered using SLAF and BSR-seq. *BMC Plant Biol* 22(1)**,** 83. doi: 10.1186/s12870-022-03448-5.

Wiersma, A.T., Pulman, J.A., Brown, L.K., Cowger, C., and Olson, E.L. (2017). Identification of Pm58 from Aegilops tauschii. *Theoretical and Applied Genetics* 130(6)**,** 1123-1133. doi: 10.1007/s00122-017-2874-8.

Wu, Q., Chen, Y., Li, B., Li, J., Zhang, P., Xie, J., et al. (2022). Functional characterization of powdery mildew resistance gene MlIW172, a new Pm60 allele and its allelic variation in wild emmer wheat. *J Genet Genomics*. doi: 10.1016/j.jgg.2022.01.010.

Xiao, M., Song, F., Jiao, J., Wang, X., Xu, H., and Li, H. (2013). Identification of the gene *Pm47* on chromosome 7BS conferring resistance to powdery mildew in the Chinese wheat landrace Hongyanglazi. *Theoretical and Applied Genetics* 126(5)**,** 1397-1403. doi: 10.1007/s00122-013-2060-6.

Xu, H., Yao, G., Xiong, L., Yang, L., Jiang, Y., Fu, B., et al. (2008). Identification and mapping of *Pm2026*: a recessive powdery mildew resistance gene in an einkorn (*Triticum monococcum* L.) accession. *Theoretical and Applied Genetics* 117(4)**,** 471-477. doi: 10.1007/s00122-008-0791-6.

Xu, H., Yi, Y., Ma, P., Qie, Y., Fu, X., Xu, Y., et al. (2015). Molecular tagging of a new broad-spectrum powdery mildew resistance allele Pm2c in Chinese wheat landrace Niaomai. *Theoretical and Applied Genetics* 128(10)**,** 2077-2084. doi: 10.1007/s00122-015-2568-z.

Xu, W., Li, C., Hu, L., Wang, H., Dong, H., Zhang, J., et al. (2011). Identification and molecular mapping of *PmHNK54:* a novel powdery mildew resistance gene in common wheat. *Plant Breeding* 130(6)**,** 603-607. doi: 10.1111/j.1439-0523.2011.01882.x.

Xu, W.G., Li, C.X., Hu, L., Zhang, L., Zhang, J.Z., Dong, H.B., et al. (2010). Molecular mapping of powdery mildew resistance gene *PmHNK* in winter wheat (*Triticum aestivum* L.) cultivar Zhoumai 22. *Mol Breed* 26(1)**,** 31-38. doi: 10.1007/s11032-009-9374-8.

Xu, X., Li, G., Cowger, C., Bai, G., Carver, B.F., Bian, R., et al. (2023). Identification of a novel Pm65 allele conferring a wide spectrum of resistance to powdery mildew in wheat accession PI 351817. *Phytopathology*. doi: 10.1094/PHYTO-01-23-0032-R.

Xue, F., Wang, C., Li, C., Duan, X., Zhou, Y., Zhao, N., et al. (2012). Molecular mapping of a powdery mildew resistance gene in common wheat landrace Baihulu and its allelism with *Pm24*. *Theoretical and Applied Genetics* 125(7)**,** 1425-1432. doi: 10.1007/s00122-012-1923-6.

Yahiaoui, N., Kaur, N., and Keller, B. (2009). Independent evolution of functional *Pm3* resistance genes in wild tetraploid wheat and domesticated bread wheat. *Plant J* 57(5)**,** 846-856. doi: 10.1111/j.1365-313X.2008.03731.x.

Yao, G., Zhang, J., Yang, L., Xu, H., Jiang, Y., Xiong, L., et al. (2007). Genetic mapping of two powdery mildew resistance genes in einkorn (*Triticum monococcum* L.) accessions. *Theoretical and Applied Genetics* 114(2)**,** 351-358. doi: 10.1007/s00122-006-0438-4.

Yin, G., Li, G., He, Z., Liu, J., Wang, H., and Xia, X. (2009). Molecular mapping of powdery mildew resistance gene in wheat cultivar Jimai 22. *Acta Agron Sin* 35(8)**,** 1425-1431.

Zeller, F.J., Kong, L., Hartl, L., Mohler, V., and Hsam, S.L.K. (2002). Chromosomal location of genes for resistance to powdery mildew in common wheat (*Triticum aestivum* L. em Thell.). *Euphytica* 123(2)**,** 187-194. doi: 10.1023/A:1014944619304.

Zhang, H., Guan, H., Li, J., Zhu, J., Xie, C., Zhou, Y., et al. (2010). Genetic and comparative genomics mapping reveals that a powdery mildew resistance gene *Ml3D232* originating from wild emmer co-segregates with an NBS-LRR analog in common wheat (*Triticum aestivum* L.). *Theoretical and Applied Genetics* 121(8)**,** 1613-1621. doi: 10.1007/s00122-010-1414-6.

Zhang, W., Yu, Z., Wang, D., Xiao, L., Su, F., Mu, Y., et al. (2023). Characterization and identification of the powdery mildew resistance gene in wheat breeding line ShiCG15-009. *BMC Plant Biol* 23(1)**,** 113. doi: 10.1186/s12870-023-04132-y.

Zhao, Z., Sun, H., Song, W., Lu, M., Huang, J., Wu, L., et al. (2013). Genetic analysis and detection of the gene *MlLX99* on chromosome 2BL conferring resistance to powdery mildew in the wheat cultivar Liangxing 99. *Theoretical and Applied Genetics* 126(12)**,** 3081-3089. doi: 10.1007/s00122-013-2194-6.

Zhou, R., Zhu, Z., Kong, X., Huo, N., Tian, Q., Li, P., et al. (2005). Development of wheat near-isogenic lines for powdery mildew resistance. *Theor Appl Genet* 110(4)**,** 640-648. doi: 10.1007/s00122-004-1889-0.

Zhu, K., Li, M., Wu, H., Zhang, D., Dong, L., Wu, Q., et al. (2022). Fine mapping of powdery mildew resistance gene MlWE74 derived from wild emmer wheat (Triticum turgidum ssp. dicoccoides) in an NBS-LRR gene cluster. *Theor Appl Genet*. doi: 10.1007/s00122-021-04027-2.

Zhu, Z., Zhou, R., Kong, X., Dong, Y., and Jia, J. (2005). Microsatellite markers linked to 2 powdery mildew resistance genes introgressed from *Triticum carthlicum* accession PS5 into common wheat. *Genome* 48(4)**,** 585-590. doi: 10.1139/g05-016.

Zhu, Z., Zhou, R., Kong, X., Dong, Y., and Jia, J. (2006). Microsatellite marker identification of a *Triticum Aestivum -Aegilops Umbellulata* substitution line with powdery mildew resistance. *Euphytica* 150(1-2)**,** 149-153. doi: 10.1007/s10681-006-9103-x.

Zou, S.H., Wang, H., Li, Y.W., Kong, Z.S., and Tang, D.Z. (2018). The NB-LRR gene Pm60 confers powdery mildew resistance in wheat. *New Phytologist* 218(1)**,** 298-309. doi: 10.1111/nph.14964.
